# Association between dietary nutrient intake and sleep disorders in hypertensive patients: a cross-sectional study of NHANES, 2005–2020

**DOI:** 10.3389/fnut.2025.1586530

**Published:** 2025-06-04

**Authors:** Aokai Tian, Yafang Zheng, Jing Jin, Chunyuan Huang

**Affiliations:** Liaoning University of Traditional Chinese Medicine, Shenyang, Liaoning, China

**Keywords:** hypertension, dietary nutrition, sleep disorders, NHANES, cross-sectional studies.

## Abstract

**Background:**

Dietary nutrient intake is critical for healthy sleep patterns, but studies in hypertensive patients are lacking. This study examines the connection between hypertension patients’ and their dietary nutrient intake.

**Methods:**

Adults with complete data on hypertension, dietary nutrient intake, and sleep disorders from the 2005 to 2020 National Health and Nutrition Examination Survey (NHANES) were used. The association between dietary nutrient intake and sleep disorders in hypertensive patients was assessed by multivariable logistic regression, smoothed curve fitting, subgroup analyses, interaction tests, and threshold effect analyses.

**Results:**

There were altogether 15,871 subjects included in this work, with 5,791 individuals presenting with sleep disorders. The positive associations of dietary sugar, fat, and calcium intake with sleep disorders remained stable in fully adjusted models. Analyses of subgroups indicated that the link between sugar consumption and sleep disorders was affected by both gender and diabetes, while the connection between fat consumption and sleep disorders was influenced by gender alone. In female patients, a U-shaped association was observed between dietary sugar, fat intake, and sleep disorders, with inflection points of 140 and 66, respectively. Finally, the relationships between dietary sugar, fat, and calcium intake and various types of sleep disorders were further analyzed. The findings indicated that the intake of dietary sugar was correlated with other types of sleep, and dietary calcium intake was correlated with restless legs syndrome.

**Conclusion:**

Our results indicate that the increased risk of sleep disorders in hypertensive patients may be associated with increased dietary sugar, fat, and calcium intake.

## 1 Introduction

Sleep has a significant role in human physiological activity. However, sleep disorders are prevalent globally, with approximately 30–50% of the population suffering from varying degrees of sleep disorders (1, 2), and their incidence is increasing year by year. Impacting people’s quality of life and increasing their risk for chronic conditions like diabetes, obesity, and cardiovascular disease (3). Numerous physical, psychological, environmental, and social factors have been linked to sleep disturbances, according to research (4). Recent studies have investigated the nexus between dietary intake and sleep patterns, with particular attention to the impact of specific nutrients on sleep quality and architecture (5). Sleep disorders are characterized by the chronic inability to get enough sleep, leading to conditions such as insomnia, obstructive sleep apnea, narcolepsy, and restless legs syndrome (6).

Hypertension is a common chronic disease with a global prevalence of approximately 34 per cent among adults aged 30–79 years (7). It is often seen alongside sleep problems like insomnia and obstructive sleep apnea (8). A bidirectional association exists between hypertension and sleep disorders: On the one hand, sleep deprivation or compromised sleep quality may precipitate elevated blood pressure levels (9, 10); On the other hand, hypertension itself may further exacerbate sleep problems by affecting the autonomic nervous system and vascular functioning (8). In addition, hypertension is frequently linked to other metabolic disorders, like obesity and diabetes mellitus, which may also indirectly impair sleep quality (11–13).

Dietary intake is a critical determinant of sleep quality. The extant research has proven that the intake of certain nutrients is inextricably connected to sleep quality. For example, a diet high in sugar can induce fluctuations in blood sugar and affect sleep stability (12, 14–16); High-fat diets may disrupt sleep rhythms by affecting the neuroendocrine system (3, 5, 17–19); Calcium ions play an important role in nerve conduction (20, 21), muscle relaxation (22, 23) and sleep regulation (24–26). Calcium affects sleep onset and maintenance by regulating neurotransmitter release and muscle contraction.

Most of the current studies on the relationship between diet and sleep are based on the general population and fail to adequately consider the special physiopathological characteristics of hypertensive patients, such as autonomic dysfunction and vascular endothelial dysfunction, which may have a regulatory effect on the diet-sleep relationship. Moreover, there is a lack of systematic research on how dietary components affect sleep quality through blood pressure regulatory pathways (e.g., renin-angiotensin system, oxidative stress, etc.) and whether this effect differs from that of the normal population. The present study investigates the possible relation between dietary nutritional intake and the prevalence of sleep disorders in hypertensive individuals, with the NHANES database.

The aim of this study was to systematically assess the specific association between dietary nutritional intake and sleep disorders in hypertensive patients, and to provide an evidence-based basis for the development of personalized nutritional intervention strategies that take into account blood pressure control and sleep improvement in hypertensive patients. The reasons for conducting this study include the significantly higher prevalence of sleep disorders in hypertensive patients than in the general population and the limited availability of targeted interventions. Current dietary guidelines for hypertension focus primarily on blood pressure control, with less consideration given to the comprehensive impact on sleep quality. With this study, we expect to fill the knowledge gap in the study of the relationship between diet and sleep in the special population of hypertension, and to provide more precise and comprehensive guidance on lifestyle interventions for this high-burden population.

## 2 Materials and methods

### 2.1 Objects of study

**FIGURE 1:**
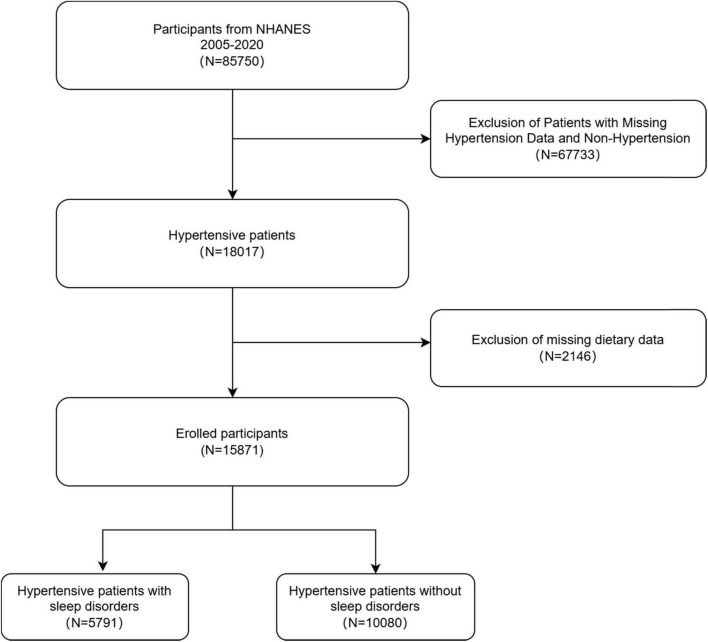
Flowchart for inclusion and exclusion of participants in the analysis.

Data used in the present study were obtained in the National Health and Nutrition Examination Survey (NHANES), an ongoing survey carried out by the National Centre for Health Statistics (NCHS) covering health and nutritional status data for the U.S. It utilizes stratified, multistage probability sampling and is conducted at 2-year intervals to ensure sample representativeness. The research protocols and consent procedures were authorized by the NCHS Ethics Committee. All data collected during the course of the survey are available to the public at the following address: https://www.cdc.gov/nchs/nhanes/. Data from the NHANES survey on hypertension, dietary nutrient intake, and sleep disorders during 2005–2020.3 were selected for this study. Initially, 85,750 eligible participants aged ≥ 18 years were recruited. After excluding 67,733 participants due to incomplete data on hypertension status (either missing or non-hypertensive) and 2,146 participants with incomplete dietary data, a final sample of 15,871 individuals was included in the study. Among these, 5,791 participants had sleep disorders, while 10,080 did not. The selection process for the study population is illustrated in Figure 1.

### 2.2 Assessment of hypertension and sleep disorders

Data on hypertension and sleep disorders were collected through self-reported responses from the Medical Condition Questionnaire (MCQ). Hypertension was identified by asking participants if they had ever been informed by a healthcare professional about having high blood pressure. Individuals who responded in the affirmative were designated as hypertensive, whereas those who responded in the negative were categorized as non-hypertensive. Sleep disorders were assessed using the SLQ050 questionnaire from the NHANES survey, which enquired if participants had disclosed to their healthcare provider about having a sleep disorder (yes or no). Respondents who acknowledged the presence of a sleep disorder were categorized as such, whereas those who denied the existence of a sleep disorder were categorized as not having one. Additionally, participants who self-reported specific types of sleep disorders such as sleep apnea (SLQ070A), insomnia (SLQ070B), restless leg syndrome (SLQ070C), and other sleep disorders (SLQ070D) were included for further analysis based on the SLQ070 sleep disorder questionnaire. Due to constraints in the database, only participants with available data on sleep disorder type from NHANES 2005 to 2008 were included in the analysis.

### 2.3 Dietary nutrient intake

NHANES participants were given the opportunity to undergo two detailed 24-h dietary recall interviews for the purpose of assessing their intake of various nutrients. The initial interview was undertaken at a mobile examination center (MEC), where trained interviewers meticulously adhered to a standardized protocol to obtain exhaustive information regarding the foods consumed by the participant in the preceding 24 h. A second interview, to be conducted via telephone, was scheduled to take place from 3 to 10 days hence. The purpose of this interview was to gather additional dietary data, thereby enabling a more accurate assessment of nutrient intake. The daily dietary nutrient intake of each participant in the study was calculated by averaging the information provided in the two dietary recall interviews. The dietary survey encompassed a wide range of 31 nutrients sourced from the NHANES database, including energy (kcal), protein (g), carbohydrate (g), total sugars (g), dietary fiber (g), total fat (g), cholesterol (mg), vitamin E (mg), retinol (mcg), vitamin A (mcg), α-carotene (mcg), β-carotene (mcg), β-cryptoxanthin (mcg), lycopene (mcg), vitamin B1 (mg), vitamin B2 (mg), niacin (mg), vitamin B6 (mg), folic acid (mcg), vitamin B12 (mcg), vitamin C (mg), vitamin K (mg), calcium (mg), phosphorus (mg), magnesium (mg), iron (mg), zinc (mg), copper (mg), sodium (mg), potassium (mg), and selenium (mcg). Through this thorough and meticulous dietary assessment process, researchers were able to gather valuable information on the nutrient intake of NHANES participants, enabling a more complete understanding of their dietary habits and nutritional status.

### 2.4 Covariates

This study examined a range of variables, including age, gender, ethnicity, education, body mass index (BMI), marital status, smoking, presence of diabetes, total cholesterol levels, history of coronary heart disease, history of stroke, frequency of alcohol consumption, and family poverty-to-income ratio (Family PIR). Race/ethnicity was classified into five groups: Mexican American, Other Hispanic, Non-Hispanic White, Non-Hispanic Black, and Other. Educational attainment was categorized as less than high school, high school graduate, and more than high school. BMI was divided into three categories: < 25 kg/m^2^ (normal weight), 25–29.9 kg/m^2^ (overweight), and ≥ 30 kg/m^2^ (obese). Marital status was grouped as married/cohabiting, divorced/widowed/separated, and unmarried. Smoking was categorized into never or smoking; 100 cigarettes during a lifetime was deemed to be never smoking, while ≥ 100 cigarettes during a lifetime was deemed to be smoking. Alcohol consumption is used to denote the frequency with which participants imbibed alcohol during the preceding 12 months. Information on diabetes, coronary heart disease, and stroke was obtained from self-reported responses to the questionnaire.

### 2.5 Statistical analysis

*P* < 0.05 stood for significance using R (version 4.1.3) and EmpowerStats (version 2.0). Continuous data were represented by mean ± standard deviation (SD), while categorical data were represented by percentages. We compared differences in sleep disorder vs. non-sleep disorder groups by chi-square tests (categorical data) or *t*-tests (continuous data). Ratio ratios (OR) with 95% confidence intervals (CI) of dietary nutritional intake and sleep disorders in hypertensive patients were generated by multivariate logistic regression. There were three models generated through multivariate testing: model 1 was not adjusted for any variable; in model 2, age, sex, and ethnicity were adjusted; and in model 3, every variable in Table 1 was adjusted. Non-linear correlations between the dietary nutritional intake and sleep disorders in hypertensive patients were assessed using smoothed curve fitting. In addition, subgroup analyses based on age, sex, ethnicity, education, marriage, BMI, smoking, diabetes mellitus, coronary heart disease, and stroke, which were factors, were also tested for heterogeneity of associations between subgroups by adding an interaction term, and, for missing values, either plurality interpolation (categorical data) or median interpolation (continuous data) was used.

**TABLE 1:** Baseline characteristics of hypertensive patients were described according to the two groups (sleep-disordered and non-sleep-disordered).

Baseline characteristics	Total	Sleep disorder	Non-sleep disorder	*P*-value
	*N* = 15,871	*N* = 5,791	*N* = 10,080	
Age (years)	59.32 ± 15.48	58.75 ± 14.45	59.64 ± 16.04	< 0.001***
Gender, *n* (%)				< 0.001***
Male	7,694 (48.48%)	2,484 (42.89%)	5,210 (51.69%)	
Female	8,177 (51.52%)	3,307 (57.11%)	4,870 (48.31%)	
Race, *n* (%)				< 0.001***
Mexican American	1,747 (11.01%)	516 (8.91%)	1,231 (12.21%)	
Other Hispanic	1,367 (8.61%)	506 (8.74%)	861 (8.54%)	
Non-Hispanic White	6,761 (42.60%)	2,710 (46.80%)	4,051 (40.19%)	
Non-Hispanic Black	4,559 (28.73%)	1,616 (27.91%)	2,943 (29.20%)	
Other races	1,437 (9.05%)	443 (7.65%)	994 (9.86%)	
Education level, *n* (%)				0.009**
>High school	4,024 (25.35%)	1,388 (23.97%)	2,636 (26.15%)	
High school	3,898 (24.56%)	1,456 (25.14%)	2,442 (24.23%)	
>High school	7,949 (50.09%)	2,947 (50.89%)	5,002 (49.62%)	
Marital status, *n* (%)				< 0.001***
Married/cohabiting	9,155 (57.68%)	3,099 (53.51%)	6,056 (60.08%)	
Divorced/widowed/ separated	5,353 (33.73%)	2,170 (37.47%)	3,183 (31.58%)	
Never married	1,363 (8.59%)	522 (9.01%)	841 (8.34%)	
BMI (kg/m^2^), *n* (%)				< 0.001***
<25	2,716 (17.11%)	840 (14.51%)	1,876 (18.61%)	
≥ 25, <30	4,906 (30.91%)	1,575 (27.20%)	3,331 (33.05%)	
≥30	8,249 (51.98%)	3,376 (58.30%)	4,873 (48.34%)	
Smoking, *n* (%)				< 0.001***
Ever	7,815 (49.24%)	3,144 (54.29%)	4,671 (46.34%)	
Never	8,056 (50.76%)	2,647 (45.71%)	5,409 (53.66%)	
Diabetes, *n* (%)				< 0.001***
Yes	4,023 (25.35%)	1,671 (28.86%)	2,352 (23.33%)	
No	11,848 (74.65%)	4,120 (71.14%)	7,728 (76.67%)	
Coronary heart disease, *n* (%)				< 0.001***
Yes	1,368 (8.62%)	590 (10.19%)	778 (7.72%)	
No	14,503 (91.38%)	5,201 (89.81%)	9,302 (92.28%)	
Stroke,*n* (%)				< 0.001***
Yes	1,353 (8.52%)	637 (11.00%)	716 (7.10%)	
No	14,518 (91.48%)	5,154 (89.00%)	9,364 (92.90%)	
Family PIR (mean ± SD)	2.43 ± 1.52	2.33 ± 1.52	2.48 ± 1.51	< 0.001***
Total cholesterol (mg)	190.00 ± 42.25	189.82 ± 42.99	190.10 ± 41.82	0.410
Alcohol consumption (mean ± SD)	4.48 ± 32.46	4.44 ± 30.35	4.50 ± 33.62	0.319

PIR, Ratio of family income to poverty; BMI, body mass index. *p < 0.05, **p < 0.01, ***p < 0.001; ap < 0.05 was considered statistically significant.

## 3 Results

### 3.1 Baseline characteristics of hypertensive patients

There were 15,871 subjects in this work, and the average age was 59.32 ± 15.48 years. Among the participants, 48.48% were male and 51.52% were female. The majority of the individuals were non-Hispanic White who had completed high school education or higher; 57.68% were married/cohabiting, 51.98% had a BMI ≥ 30, smokers *vs*. non-smokers was close to 1:1, one-quarter had diabetes, and both coronary artery disease and stroke accounted for a smaller percentage. In addition, there were 5,791 sleep-disordered participants (42.89% male and 57.11% female). Compared with the group of non-sleep-disordered participants, sleep-disordered participants tended to be younger, have lower total cholesterol, drink less alcohol, have lower household income, be married and cohabiting less, have lower BMI, smoke less, have less diabetes mellitus, have less coronary artery disease, and have fewer strokes (Table 1).

### 3.2 Baseline characteristics of dietary intake levels in hypertensive patients

Upon analyzing the dietary nutrient intake of both groups of participants in Table 2, those with sleep disorders were found to have higher levels of total dietary sugar, total fat, retinol, and calcium (*p* < 0.05) in comparison with individuals without sleep disorders. Conversely, dietary fiber, α-carotene, β-carotene, lycopene, vitamin B6, vitamin C, copper, and potassium were lower (*p* < 0.05). However, no substantial disparities were detected in the consumption of additional dietary nutrients (*p* > 0.05).

**TABLE 2:** Baseline characteristics of dietary intake levels in hypertensive patients in both groups (sleep-disordered and non-sleep-disordered groups).

Dietary nutrition	Total	Sleep disorder	Non-sleep disorder	*P*-value
	*N* = 15,871	*N* = 5,791	*N* = 10,080	
Energy, kcal	1810.59 ± 807.90	1827.27 ± 850.12	1801.01 ± 782.50	0.605
Protein, g	71.04 ± 33.48	70.79 ± 34.39	71.18 ± 32.95	0.107
Carbohydrate, g	216.15 ± 101.28	217.86 ± 107.38	215.18 ± 97.60	0.890
**Total sugars, g**	94.59 ± 60.38	98.08 ± 65.64	92.58 ± 57.04	< 0.001***
**Dietary fiber, g**	14.93 ± 8.48	14.51 ± 8.25	15.17 ± 8.60	< 0.001***
**Total fat, g**	70.81 ± 37.66	72.22 ± 39.49	70.00 ± 36.55	**0.018**
Cholesterol, mg	270.96 ± 186.76	274.88 ± 193.84	268.71 ± 182.55	0.238
Vitamin E, mg	7.29 ± 4.93	7.42 ± 5.11	7.22 ± 4.82	0.219
**Retinol, mcg**	382.11 ± 448.50	391.02 ± 412.10	376.99 ± 468.07	< 0.001***
Vitamin A, mcg	576.29 ± 552.50	575.99 ± 534.05	576.46 ± 562.85	0.834
**Alpha-carotene, mcg**	365.51 ± 932.71	354.28 ± 1157.92	371.96 ± 774.24	< 0.001***
**Beta-carotene, mcg**	2104.11 ± 3282.55	2001.06 ± 3411.56	2163.32 ± 3204.76	< 0.001***
Beta-cryptoxanthin, mcg	92.48 ± 222.14	88.16 ± 172.82	94.96 ± 246.02	0.191
**Lycopene, mcg**	4216.50 ± 6443.85	4165.64 ± 6648.18	4245.72 ± 6323.62	0.040*
Vitamin B1, mg	1.40 ± 0.73	1.40 ± 0.76	1.40 ± 0.71	0.251
Vitamin B2, mg	1.81 ± 1.02	1.85 ± 1.10	1.79 ± 0.97	0.075
Niacin, mg	21.90 ± 11.88	21.83 ± 12.21	21.94 ± 11.69	0.078
**Vitamin B6, mg**	1.81 ± 1.25	1.79 ± 1.29	1.81 ± 1.22	0.004**
Folic acid, mcg	151.71 ± 134.00	152.43 ± 140.81	151.30 ± 129.94	0.209
Vitamin B12, mcg	4.46 ± 4.98	4.47 ± 4.60	4.46 ± 5.19	0.775
**Vitamin C, mg**	75.81 ± 71.27	73.81 ± 71.55	76.96 ± 71.09	< 0.001***
Vitamin K, mg	105.89 ± 136.09	102.43 ± 121.13	107.87 ± 143.95	0.131
**Calcium, mg**	806.44 ± 456.26	826.58 ± 479.03	794.87 ± 442.26	< 0.001***
Phosphorus, mg	1172.62 ± 544.43	1179.74 ± 570.14	1168.53 ± 529.07	0.996
Magnesium, mg	259.91 ± 123.64	258.87 ± 126.62	260.51 ± 121.90	0.055
Iron, mg	13.02 ± 7.08	12.98 ± 7.27	13.05 ± 6.97	0.151
Zinc, mg	9.82 ± 5.97	9.81 ± 5.93	9.82 ± 5.99	0.402
**Copper, mg**	1.11 ± 0.83	1.10 ± 0.76	1.12 ± 0.87	0.003**
Sodium, mg	2998.83 ± 1439.47	3006.93 ± 1483.86	2994.17 ± 1413.39	0.664
**Potassium, mg**	2341.92 ± 1067.22	2330.49 ± 1098.11	2348.49 ± 1049.06	0.035*
Selenium, mcg	99.22 ± 50.07	98.78 ± 50.64	99.48 ± 49.74	0.208

*p < 0.05, **p < 0.01, ***p < 0.001; ap < 0.05 was considered statistically significant. Significant values are in bold.

### 3.3 Connection between dietary nutritional intake and sleep disorders in hypertensive patients

The effect value was magnified 100-fold using the dietary nutrient intake value/100 because the effect value was not significant. Multivariate regression analysis of dietary nutrient intake/100 and sleep disturbance in hypertensive patients is shown in Table 3. In model 1, total dietary sugar, total fat, and calcium intake were positively associated with sleep disturbance (*p* < 0.01). Three models were created after adjusting for relevant confounding variables. The findings indicated that in models 2 and 3, the positive correlation between total dietary sugar, total fat, and calcium intake and sleep disturbance remained stable (*p* < 0.01). However, the correlations between dietary fiber, retinol, α-carotene, β-carotene, lycopene, vitamin B6, vitamin C, copper, and potassium intake levels and sleep disorders were no statistically significant differences (*p* > 0.05).

**TABLE 3:** Relationship between dietary nutritional intake and sleep disorders in hypertensive patients.

Exposure	Model 1 [OR (95% CI)]	*P*-value	Model 2 [OR (95% CI)]	*P*-value	Model 3 [OR (95% CI)]	*P*-value
**Total sugars/100**	**1.16 (1.10, 1.22)**	< 0.0001***	**1.16 (1.10, 1.23)**	< 0.0001***	**1.19 (1.13, 1.26)**	< 0.0001***
Dietary fiber/100	0.39 (0.27, 0.58)	< 0.0001***	0.61 (0.41, 0.92)	0.0172	0.93 (0.61, 1.41)	0.7355
**Total fat/100**	**1.17 (1.07, 1.27)**	0.0004**	**1.25 (1.14, 1.37)**	< 0.0001***	**1.21 (1.10, 1.33)**	< 0.0001***
Retinol/100	1.01 (1.00, 1.01)	0.0599	1.01 (1.00, 1.01)	0.0887	1.01 (1.00, 1.01)	0.0941
Alpha-carotene/100	1.00 (0.99, 1.00)	0.2539	1.00 (0.99, 1.00)	0.3490	1.00 (1.00, 1.00)	0.7827
Beta-carotene/100	1.00 (1.00, 1.00)	0.0028**	1.00 (1.00, 1.00)	0.0066**	1.00 (1.00, 1.00)	0.1769
Lycopene/100	1.00 (1.00, 1.00)	0.4511	1.00 (1.00, 1.00)	0.5726	1.00 (1.00, 1.00)	0.8744
Vitamin B6/100	0.33 (0.02, 4.53)	0.4073	1.74 (0.12, 25.15)	0.6826	5.62 (0.37, 85.26)	0.2130
Vitamin C/100	0.94 (0.90, 0.98)	0.0074**	0.96 (0.92, 1.01)	0.0999	1.00 (0.95, 1.05)	0.9703
**Calcium/100**	**1.02 (1.01, 1.02)**	< 0.0001***	**1.02 (1.01, 1.02)**	< 0.0001***	**1.02 (1.01, 1.03)**	< 0.0001***
Copper/100	0.02 (0.00, 1.28)	0.0654	0.16 (0.00, 9.18)	0.3734	1.03 (0.02, 59.05)	0.9884
Potassium/100	1.00 (1.00, 1.00)	0.3062	1.00 (1.00, 1.00)	0.5255	1.00 (1.00, 1.01)	0.0466*
Total sugars (quartile)						
Quartile 1	Reference		Reference		Reference	
Quartile 2	0.98 (0.89, 1.07)	0.6441	0.97 (0.88, 1.06)	0.4601	0.99 (0.90, 1.08)	0.7654
Quartile 3	1.05 (0.96, 1.15)	0.2820	1.04 (0.95, 1.14)	0.4408	1.09 (0.99, 1.20)	0.0636
Quartile 4	1.17 (1.07, 1.28)	0.0008**	1.16 (1.06, 1.28)	0.0017**	1.23 (1.11, 1.35)	< 0.0001***
P for trend	1.15 (1.07, 1.24)	< 0.0001***	1.15 (1.07, 1.24)	0.0002***	1.20 (1.12, 1.30)	< 0.0001***
Total fat (quartile)						
Quartile 1	Reference		Reference		Reference	
Quartile 2	1.03 (0.94, 1.13)	0.4672	1.02 (0.93, 1.12)	0.6197	1.03 (0.93, 1.13)	0.5778
Quartile 3	0.99 (0.91, 1.09)	0.8935	1.00 (0.91, 1.09)	0.9413	0.99 (0.90, 1.09)	0.7786
Quartile 4	1.13 (1.03, 1.24)	0.0081**	1.19 (1.08, 1.31)	0.0003***	1.17 (1.06, 1.29)	0.0019**
P for trend	1.15 (1.03, 1.28)	0.0124*	1.23 (1.10, 1.38)	0.0004***	1.20 (1.07, 1.35)	0.0025**
Calcium (quartile)						
Quartile 1	Reference		Reference		Reference	
Quartile 2	1.00 (0.92, 1.10)	0.9330	0.99 (0.90, 1.09)	0.8135	1.00 (0.91, 1.10)	0.9767
Quartile 3	1.05 (0.96, 1.15)	0.2642	1.04 (0.95, 1.14)	0.3742	1.06 (0.97, 1.17)	0.2141
Quartile 4	1.13 (1.03, 1.24)	0.0087**	1.14 (1.04, 1.25)	0.0073**	1.18 (1.07, 1.30)	0.0010**
P for trend	1.01 (1.00, 1.02)	0.0036**	1.02 (1.01, 1.02)	0.0024**	1.02 (1.01, 1.02)	0.0003**

Model 1, no covariates were adjusted; Model 2, age, gender, and race were adjusted; Model 3, gender, age, race, Education level, Marital status; PIR; BMI; Total cholesterol; Alcohol consumption; smoking; Diabetes; Coronary heart disease and stroke were adjusted. 95% CI, 95% confidence interval; OR, odds ratio; *p < 0.05, **p < 0.01, ***p < 0.001; ap < 0.05 was considered statistically significant. Significant values are in bold.

In model 1, unadjusted for covariates, total dietary sugar intake was positively correlated with sleep disturbance (OR = 1.16; 95% CI: 1.10, 1.22; *p* < 0.0001), indicating that for every 1 g increase in total dietary sugar intake, sleep disturbance increased by 16%. Total dietary fat intake was positively correlated with sleep disorders (OR = 1.17; 95% CI: 1.07, 1.27; *p* = 0.0004), indicating a 17% increase in sleep disorders per 1 g increase in total dietary fat intake. Dietary calcium intake was positively correlated with sleep disorders (OR = 1.02; 95% CI: 1.01, 1.02; *p* < 0.0001), indicating a 2% increase in sleep disorders for each 1 mg increase in dietary calcium intake. After adjusting for age, gender, and ethnicity in Model 2, the results remained consistent with those of Model 1. Total dietary sugar intake was still positively associated with sleep disorders (OR = 1.16; 95% CI: 1.10-1.23; *p* < 0.0001), indicating a 16% increase in the likelihood of sleep disorders for each 1 g increase in total dietary sugar intake. Similarly, total dietary fat intake showed a positive association with sleep disorders (OR = 1.25; 95% CI: 1.14, 1.37; *p* < 0.0001), suggesting a 25% increase in sleep disorders per 1 g increase in total dietary fat intake. Additionally, dietary calcium intake remained positively associated with sleep disorders (OR = 1.02; 95% CI: 1.01, 1.02; *p* < 0.0001), indicating a 2% increase in the likelihood of experiencing sleep disorders for every 1 mg increase in dietary calcium intake. Further adjustments were made in Model 3 for gender, age, race, education level, marital status, PIR, BMI, total cholesterol, alcohol consumption, smoking, diabetes, coronary heart disease, and stroke. The results in Model 3 showed that the positive associations of total dietary sugar, total fat, and calcium intake with sleep disorders remained significant. Total dietary sugar intake was associated with sleep disorders at an OR of 1.19 (95% CI: 1.13–1.26; *p* < 0.0001), total fat intake at an OR of 1.21 (95% CI: 1.10–1.33; *p* < 0.0001), and dietary calcium intake at an OR of 1.02 (95% CI: 1.01-1.03; *p* < 0.0001). These findings suggest that for every 1-unit increase in total dietary sugar, total fat, and calcium intake, there was a 19, 21, and 2% increase in the likelihood of experiencing sleep disorders, respectively.

For sensitivity analyses, we also shifted total dietary sugar, total fat, and calcium from continuous to categorical variables (quartiles). This trend was consistent when total dietary sugar, total fat, and calcium intake were analyzed in quartiles (*p* for trend < 0.05). In model 1, the odds of sleep disorders were increased by 17, 13, and 13%, respectively, in quartile 4 compared with quartile 1. In model 2, the odds of sleep disorders in quartile 4 increased by 16, 19, and 14%, respectively, compared with quartile 1. In Model 3, the odds of sleep disorders increased by 23, 17, and 18% in Quartile 4 compared to Quartile 1, respectively. However, in models 1, 2, and 3, neither quartiles 2 nor 3 showed any statistical difference (*p* > 0.05).

### 3.4 Stratified analysis of the correlation between dietary nutritional intake and sleep disorders in hypertensive patients

In order to better understand how total dietary sugar, total fat, calcium intake, and sleep disorders are related and influenced by various factors, we conducted detailed analyses based on different demographic and health variables. These variables included age, gender, race, education, marital status, BMI, smoking habits, diabetes, coronary heart disease, and stroke. We found the correlation between total dietary sugar and sleep disorders to be influenced by both gender and diabetes, while the association between total dietary fat and sleep disorders was influenced by gender. Interestingly, we did not observe any significant interactions for the other variables we analyzed in relation to total dietary sugar, total fat, calcium intake, and sleep disorders. This suggests that factors such as age, race, education, marital status, BMI, smoking habits, coronary heart disease, and stroke did not have a significant impact on the positive correlation between total dietary sugar, total fat, calcium intake, and sleep disorders.

### 3.5 Smooth curve fitting and threshold effect analysis

**FIGURE 2:**
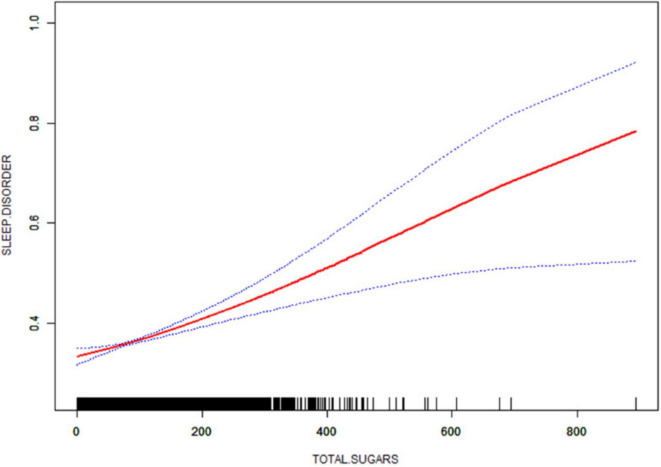
Non-linear relationship between total dietary sugar intake and sleep disorders. The solid red line indicates a smooth curve fit between the variables. The 95% confidence intervals for the fitted results are indicated by the blue bars.

**FIGURE 3:**
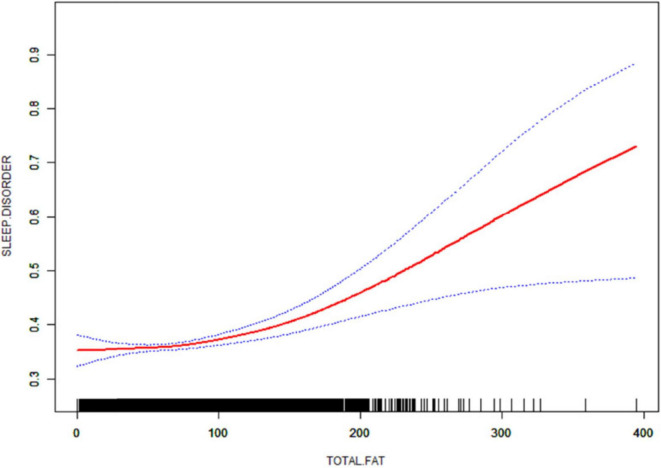
Non-linear relationship between total dietary fat intake and sleep disorders. The solid red line indicates a smooth curve fit between the variables. The 95% confidence intervals for the fitted results are indicated by the blue bars.

**FIGURE 4:**
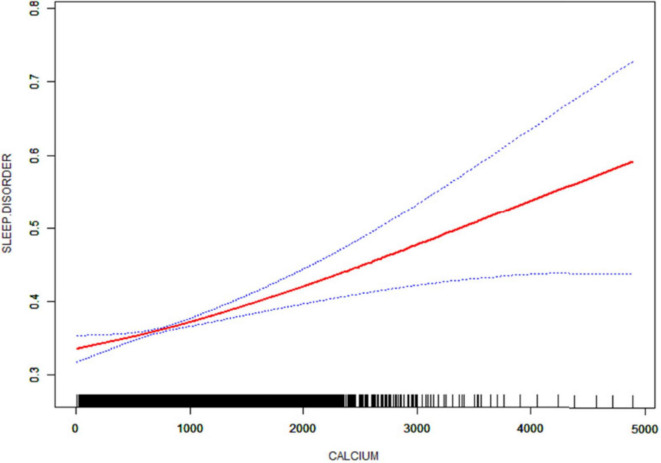
Non-linear relationship between dietary calcium intake and sleep disorders. The solid red line indicates a smooth curve fit between the variables. The 95% confidence intervals for the fitted results are indicated by the blue bars.

**FIGURE 5:**
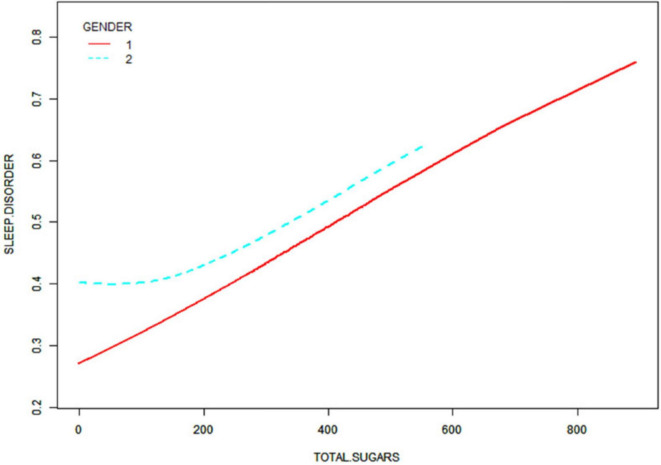
Correlation between total dietary sugar intake and sleep disorders stratified by gender.

**FIGURE 6:**
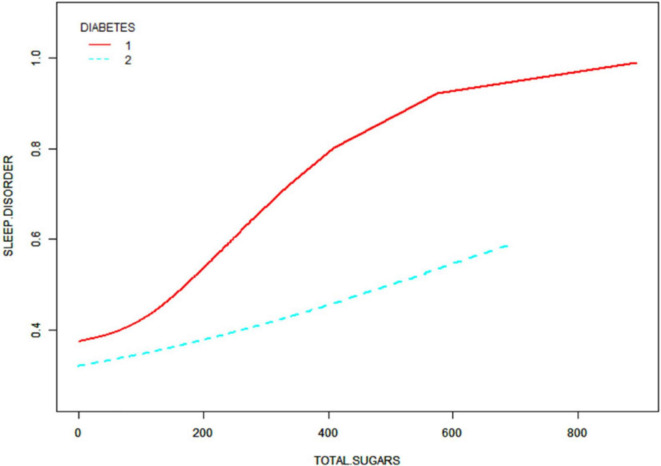
Correlation between total dietary sugar intake and sleep disorders stratified by diabetes.

**FIGURE 7:**
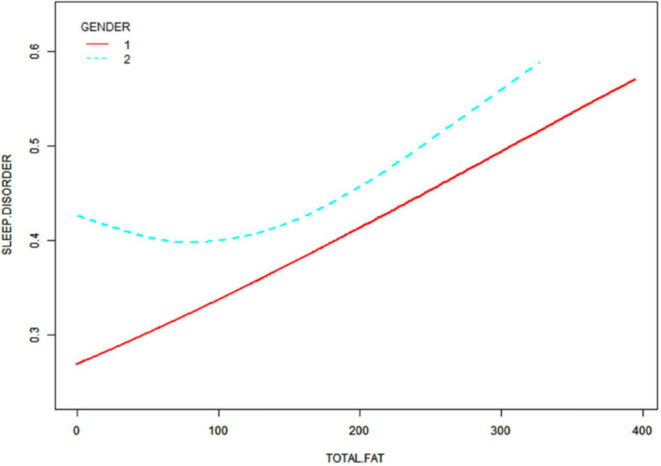
Correlation between total dietary fat intake and sleep disorders stratified by gender.

After that, non-linear associations between total dietary sugar, total fat, and calcium intake and sleep disorders were assessed using smoothed curve fitting (Figures 2–4). The findings indicated that total dietary sugar, total fat, and calcium intake were all positively associated with sleep disorders. According to Table 4, the correlation between total dietary sugar and sleep disorders was further analyzed stratified by gender and diabetes (Figures 5, 6), and the correlation between total dietary fat and sleep disorders was stratified by gender (Figure 7). We found that when stratified by sex, among women, the existence of a U-shaped connection was observed between total dietary sugar and total fat intake and sleep disorders. Among diabetic patients, the proportion of sleep disorders was low when total sugar intake was low, but the proportion increased rapidly with increasing intake and leveled off at about 600 g of total sugar intake, approaching one.

**TABLE 4:** Subgroup analysis of dietary nutrient intake and sleep disorders in hypertensive patients.

Dietary nutrient intake/100	Total sugars/100		Total fat/100		Calcium/100	
	[OR (95%CI)]	*P* for interaction	[OR (95%CI)]	*P* for interaction	[OR (95%CI)]	*P* for interaction
Age		0.5631		0.0235*		0.3381
< 60 years	1.16 (1.08, 1.24)		1.06 (0.93, 1.20)		1.01 (1.00, 1.02)	
≥ 60 years	1.20 (1.09, 1.31)		1.31 (1.14, 1.51)		1.02 (1.01, 1.03)	
**Gender, *n* (%)**		0.0016**		0.0038**		0.1687
Male	1.29 (1.20, 1.40)		1.36 (1.21, 1.54)		1.02 (1.01, 1.03)	
Female	1.07 (0.98, 1.17)		1.03 (0.88, 1.19)		1.01 (1.00, 1.02)	
Race, *n* (%)		0.7311		0.2335		0.7662
Mexican American	1.13 (0.92, 1.39)		1.09 (0.80, 1.48)		1.02 (0.99, 1.04)	
Other Hispanic	1.18 (0.96, 1.44)		1.12 (0.78, 1.62)		1.04 (1.01, 1.07)	
Non-Hispanic White	1.18 (1.08, 1.28)		1.16 (1.01, 1.34)		1.01 (1.00, 1.02)	
Non-Hispanic Black	1.26 (1.13, 1.40)		1.37 (1.15, 1.62)		1.04 (1.02, 1.06)	
Other race	1.06 (0.86, 1.32)		1.17 (0.83, 1.66)		1.00 (0.98, 1.03)	
Education level, *n* (%)		0.0580		0.1433		0.1366
<High school	1.30 (1.16, 1.45)		1.29 (1.06, 1.58)		1.03 (1.01, 1.04)	
High school	1.20 (1.08, 1.34)		1.35 (1.12, 1.62)		1.03 (1.01, 1.04)	
¿High school	1.14 (1.05, 1.23)		1.12 (0.99, 1.27)		1.01 (1.00, 1.02)	
Marital status, *n* (%)		0.3469		0.6894		0.7415
Married/cohabiting	1.16 (1.08, 1.25)		1.19 (1.05, 1.35)		1.02 (1.01, 1.03)	
Divorced/widowed/ separated	1.23 (1.12, 1.36)		1.26 (1.06, 1.48)		1.03 (1.01, 1.04)	
Never married	1.23 (1.04, 1.45)		1.22 (0.92, 1.62)		1.01 (0.99, 1.04)	
BMI (kg/m^2^)		0.3299		0.5609		0.5758
<25	1.20 (1.05, 1.38)		1.19 (0.94, 1.51)		1.02 (1.00, 1.04)	
≥ 25,<30	1.28 (1.15, 1.42)		1.32 (1.11, 1.59)		1.03 (1.01, 1.04)	
≥30	1.28 (1.15, 1.42)		1.16 (1.02, 1.31)		1.02 (1.00, 1.03)	
Smoking, *n* (%)		0.0777		0.0224*		0.0195*
Ever	1.23 (1.14, 1.32)		1.32 (1.16, 1.49)		1.03 (1.02, 1.04)	
Never	1.11 (1.01, 1.21)		1.06 (0.92, 1.22)		1.01 (1.00, 1.02)	
**Diabetes, *n* (%)**		0.0092**		0.4641		0.0839
Yes	1.40 (1.22, 1.59)		1.28 (1.07, 1.53)		1.03 (1.02, 1.05)	
No	1.15 (1.08, 1.22)		1.19 (1.06, 1.32)		1.02 (1.01, 1.02)	
Coronary heart disease, *n* (%)		0.0287*		0.2376		0.5985
Yes	1.50 (1.21, 1.85)		1.01 (0.73, 1.39)		1.01 (0.99, 1.04)	
No	1.17 (1.10, 1.24)		1.23 (1.12, 1.36)		1.02 (1.01, 1.03)	
Stroke, *n* (%)		0.6051		0.7184		0.4821
Yes	1.25 (1.02, 1.54)		1.28 (0.93, 1.75)		1.03 (1.00, 1.06)	
No	1.19 (1.12, 1.26)		1.20 (1.09, 1.32)		1.02 (1.01, 1.03)	

Gender; Age; Race; Education level; Marital status; PIR; BMI; Total cholesterol.; Alcohol consumption; smoking; Diabetes; Coronary heart disease and stroke were adjusted. Abbreviation: BMI, body mass index. 95% CI, 95% confidence interval; OR, odds ratio; *p < 0.05, **p < 0.01, ***p < 0.001; ap < 0.05 was considered statistically significant. Significant values are in bold.

Threshold effect analyses were then conducted utilizing the R programming language (version 4.1.3) in conjunction with the EmpowerStats software (version 2.0) to analyze the relationship between total dietary sugar and total fat intake and sleep disorders (Table 5), which yielded that among women, the inflection points of the non-linear connections between total dietary sugar and total fat intake and sleep disorders were 140 and 66, respectively, and the results indicated that the probability of sleep disorders reached its lowest level when the intake of total sugars was 140 g and the intake of total fats was 66 g. Effect values were magnified 100-fold using dietary nutrient intake values/100 because they were not significant.

**TABLE 5:** Application of linear regression modeling to analyze the threshold effects of total sugar and total fat intake on sleep disorders in hypertensive patients.

Exposure		Adjusted OR (95% CI) *p*-value	
Total sugars/100	Total (*N* = 15871)	Inflection point/100	0.59
		Total sugars/100<0.59	0.91 (0.67, 1.22)
		Total sugars/100 ≥ 0.59	1.23 (1.15, 1.32)
		Log likelihood ratio	0.069
	Male (*N* = 7694)	Inflection point/100	1.65
		Total sugars/100<1.65	1.34 (1.19, 1.50)
		Total sugars/100 ≥ 1.65	1.23 (1.06, 1.44)
		Log likelihood ratio	0.485
	**Female** **(*N* = 8177)**	Inflection point/100	**1.4**
		Total sugars/100<1.65	0.97 (0.85, 1.10)
		Total sugars/100 ≥ 1.65	1.33 (1.07, 1.65)
		Log likelihood ratio	0.034*
Total fat/100	Total (*N* = 15,871)	Inflection point/100	1.41
		Total sugars/100<1.41	1.11 (0.99, 1.24)
		Total sugars/100 ≥ 1.41	2.02 (1.40, 2.93)
		Log likelihood ratio	0.004**
	Male (*N* = 7,694)	Inflection point/100	1.55
		Total sugars/100<1.55	1.27 (1.10, 1.47)
		Total sugars/100 ≥ 1.55	2.04 (1.24, 3.36)
		Log likelihood ratio	0.095
	**Female** **(*N* = 8177)**	Inflection point/100	**0.66**
		Total sugars/100<0.66	0.75 (0.55, 1.03)
		Total sugars/100 ≥ 0.66	1.27 (1.00, 1.63)
		Log likelihood ratio	0.031*

Gender; Age; Race; Education level; Marital status; PIR; BMI; Total cholesterol.; Alcohol consumption; smoking; Diabetes; Coronary heart disease and stroke were adjusted. 95%CI, 95% confidence interval; OR, odds ratio; *p < 0.05, **p < 0.01, ***p < 0.001; ap < 0.05 was considered statistically significant. Significant values are in bold.

### 3.6 Association of total dietary sugar, total fat, and calcium intake with type of sleep disorder

Because the effect values were not significant, dietary nutrient intake values/100 were used to magnify the effect values by a factor of 100. Multivariate regression analysis of dietary nutrient intake/100 and type of sleep disorder in hypertensive patients is shown in Table 6. Three models were created after adjusting for relevant confounding variables. The findings indicated that in models 1, 2, and 3, the positive correlation between dietary total sugar intake and other sleep types was significant (*p* < 0.01). The positive correlations between dietary calcium intake and restless legs syndrome all remained stable (*p* < 0.05). However, there was no statistically significant connection between dietary sugar, fat, and calcium intake and the remaining types of sleep disorders (*p* > 0.05).

**TABLE 6:** Associations between total dietary sugar, total fat, calcium intake and type of sleep disorder.

Exposure	Types of sleep disorders	Model 1 [OR (95% CI)]	*P*-value	Model 2 [OR (95% CI)]	*P*-value	Model 3 [OR (95% CI)]	*P*-value
Total sugars/100	OSA (*N* = 266)	1.26 (1.04, 1.52)	0.0185*	1.11 (0.91, 1.36)	0.2975	1.23 (1.00, 1.52)	0.0532
	Insomnia (*N* = 86)	1.06 (0.75, 1.50)	0.7489	1.09 (0.76, 1.55)	0.6497	1.30 (0.92, 1.84)	0.1408
	RLS (*N* = 21)	1.00 (0.49, 2.06)	0.9910	1.02 (0.49, 2.13)	0.9483	0.99 (0.49, 2.02)	0.9872
	**Other (*N* = 50)**	1.73 (1.23, 2.43)	**0.0016** **	1.63 (1.14, 2.33)	**0.0072** **	1.75 (1.24, 2.48)	**0.0016** **
Total fat/100	OSA (*N* = 266)	2.17 (1.59, 2.94)	< 0.0001***	1.67 (1.19, 2.35)	0.0028**	1.35 (0.93, 1.95)	0.1174
	Insomnia (*N* = 86)	0.38 (0.19, 0.77)	0.0075**	0.48 (0.23, 1.01)	0.0540	0.85 (0.41, 1.74)	0.6579
	RLS (*N* = 21)	1.21 (0.39, 3.78)	0.7463	1.47 (0.43, 5.06)	0.5414	1.27 (0.34, 4.72)	0.7233
	Other (*N* = 50)	1.58 (0.78, 3.20)	0.1996	1.44 (0.67, 3.10)	0.3482	1.95 (0.93, 4.08)	0.0765
Calcium/100	OSA (*N* = 266)	1.04 (1.01, 1.06)	0.0048**	1.02 (0.99, 1.05)	0.1132	1.01 (0.98, 1.04)	0.3792
	Insomnia (*N* = 86)	0.96 (0.91, 1.01)	0.1214	0.97 (0.91, 1.02)	0.2165	1.00 (0.95, 1.06)	0.9700
	**RLS (*N* = 21)**	1.09 (1.02, 1.17)	**0.0108** *	1.11 (1.03, 1.19)	**0.0057** **	1.11 (1.03, 1.20)	**0.0042** **
	Other (*N* = 50)	1.03 (0.97, 1.09)	0.3815	1.01 (0.95, 1.07)	0.7770	1.03 (0.97, 1.09)	0.3714

Model 1, no covariates were adjusted; Model 2, age, gender, and race were adjusted; Model 3, gender; age; race; Education level; Marital status; PIR; BMI; Total cholesterol.; Alcohol consumption; smoking; Diabetes; Coronary heart disease and stroke were adjusted. OSA, obstructive sleep apnea; RLS, restless legs syndrome.95% CI, 95% confidence interval; OR, odds ratio; *p < 0.05, **p < 0.01, ***p < 0.001; ap < 0.05 was considered statistically significant. Significant values are in bold.

## 4 Discussion

A total of 15,871 patients with high blood pressure were included in the study and divided into two groups based on whether they reported experiencing sleep disturbances or not. The researchers looked at the intake of 31 different dietary nutrients, such as macronutrients, vitamins, and micronutrients. In this study, we found that dietary total sugar, total fat, and calcium intake were positively associated with sleep disturbances among hypertensive patients. Further analysis revealed that the correlation between total dietary sugar intake and sleep disorders was influenced by gender and diabetes status, while the connection between total dietary fat intake and sleep disorders was affected by gender. On the other hand, the correlation between calcium intake and sleep disturbances was not influenced by any other factors. Interestingly, it was found that there was a U-shaped relationship between total dietary sugar and fat intake and sleep disorders in female hypertensive patients, with specific points of 140 and 66, respectively. The researchers also delved deeper into the specific types of sleep disorders, like sleep apnea, insomnia, and restless legs syndrome, to see if there were any unique associations with dietary intake. The conclusions drawn from this analysis indicated that only dietary total sugar intake was associated with other sleep types, and dietary calcium intake was correlated with restless legs syndrome.

Recent studies have demonstrated a mounting scholarly interest in the impact of dietary sugar and fat intake on sleep disorders. Diets rich in sugars and fats may exert detrimental effects on sleep quality and elevate the risk of sleep disorders via multifactorial pathways. Epidemiological studies and clinical trials have consistently established that diets high in sugar and fat are closely correlated with decreased sleep quality. A substantial body of research has identified that diets high in sugar and fat are significantly correlated with prolonged sleep latency, increased nocturnal awakenings, and decreased sleep efficiency (15, 27). In addition, a diet high in sugar leads to rapid fluctuations that can disrupt the stability of sleep (27). Diets rich in fat can promote obesity, a significant risk factor for obstructive sleep apnea syndrome (OSA) (28). High-sugar and high-fat diets may also disrupt the normal sleep-wake cycle by influencing the expression of genes that regulate circadian rhythms, such as Clock and Bmal1 (29). High-sugar and high-fat diets may induce metabolic disorders like insulin resistance and inflammatory responses, which can further impact the quality of sleep. Studies suggest that chronic low-grade inflammation, often caused by diets high in sugar and fat, may disrupt sleep-regulating centers via the activation of pro-inflammatory cytokines like IL-6 and TNF-α (14). Insulin resistance may lead to blood sugar fluctuations, which in turn affects sleep stability (13). High sugar and fat intake may interfere with sleep by affecting the synthesis and release of neurotransmitters. For example, diets rich in sugar and fat can cause abnormal fluctuations in dopamine and 5-hydroxytryptamine levels, neurotransmitters that play a key role in sleep conditioning (5). Furthermore, excessive intake of sugar and fat in the diet may further interfere with sleep onset and maintenance by affecting the synthesis and release of GABA (gamma-aminobutyric acid). Recent studies has shown that consuming too much sugar and fat in the diet may change the composition of the gut microbiota, which in turn affects sleep quality (30). Dysbiosis of the gut microbiota results in abnormal metabolite production (e.g., short-chain fatty acids), thereby disrupting the synthesis of neurotransmitters (e.g., 5-hydroxytryptamine and dopamine) that are critical for sleep regulation. In addition, excessive intake of sugar and fat can elevate the production of free radicals in the system, resulting in oxidative stress (31, 32), injury to the central nervous system (33), and vascular endothelial function (34), which in turn affects sleep. High-sugar and high-fat diets can also activate the sympathetic nervous system (35–37), resulting in increased blood pressure at night (38) and disturbed sleep architecture (39, 40). Excessive sympathetic hyperexcitability can lead to frequent nocturnal arousals and diminished sleep quality. Moreover, this hyperexcitability can exacerbate instability of blood pressure at night in hypertensive patients (41), further affecting sleep. In conclusion, the association between sugar and fat intake and sleep disorders encompasses a range of mechanisms, including blood glucose fluctuations, circadian dysregulation, metabolic disorders, neurotransmitter regulation, alterations in the gut microbiome, oxidative stress, and autonomic dysfunction.

Calcium intake is significantly related to sleep disorders, as evidenced by multiple studies (42, 43). Furthermore, calcium ions have been demonstrated to exert a pivotal influence on nerve conduction and muscle contraction. Gangwisch et al. (44) showed that calcium influences the sleep-wake cycle through the regulation of neurotransmitter synthesis and release (e.g., 5-hydroxytryptamine and γ-aminobutyric acid, GABA). Calcineurin (calcium-modulated neural phosphatase) has also been identified as a critical regulator of sleep, with its activity closely associated with sleep demand (45). However, excessive calcium intake may increase neuronal excitability, leading to difficulty falling asleep or sleep disruption. Calcium may improve sleep quality by modulating the synthesis and release of melatonin. Melatonin is a crucial hormone for regulating circadian rhythms and sleep. Calcium plays a pivotal role in melatonin synthesis, as calcium-dependent enzymes such as N-acetyltransferase (NAT) are rate-limiting for melatonin production (46). There is also the possibility that calcium intake may indirectly improve sleep by inhibiting inflammatory responses and reducing oxidative stress. Zemel et al. study explored the effects of calcium intake (via dairy products) on markers of oxidative stress and inflammation. The findings indicated that calcium intake significantly reduced inflammation levels (47). Orrenius et al. study explored the role of calcium ions in regulating mitochondrial oxidative stress, suggesting that calcium may play an antioxidant role by reducing free radical production (48). The above studies suggest that calcium intake helps to reduce sleep disturbances, contrary to the results of our study. The potential explanation for this discrepancy may be that hypertensive patients often take medications such as diuretics or calcium channel blockers, which may interact with calcium intake and further affect sleep. Calcium intake may also exacerbate vascular calcification, especially in hypertensive patients, where decreased vascular elasticity may affect nocturnal blood circulation, which may in turn disrupt sleep. Finally, in this study it was learned that there is a link between calcium consumption and restless legs syndrome, and many studies have also demonstrated an association between calcium intake and restless legs syndrome. Ondo et al. study finds that calcium intake may influence the occurrence and severity of RLS (49). Allen et al. suggest that calcium intake may affect RLS symptoms through modulation of neuromuscular functions (50). In conclusion, calcium intake regulates sleep via various mechanisms, such as controlling nerve conduction, melatonin synthesis, and regulation of muscle function.

Hypertensive patients often exhibit metabolic syndrome traits such as obesity and diabetes mellitus; these comorbidities may exacerbate sleep disturbances through multiple pathways (e.g., inflammation, oxidative stress). However, dietary sugar, fat, and calcium intake may further exacerbate sleep disorders through these pathways. In addition, high sugar, fat, and calcium intake can affect autonomic function, so autonomic dysfunction in hypertensive patients may further amplify sleep disturbances in this population. Currently, the exploration of the link between dietary intake of sugar, fat, and calcium and sleep disorders, particularly among hypertensive patients, remains underexplored. Conducting such studies could fill the knowledge gap in this area.

Our study has several strengths. The extensive sample size and comprehensive adjustment for covariates bolstered the reliability and applicability of the results. However, limitations remain. Our cross-sectional design did not help determine the causality, and thus a large prospective study is warranted to further elucidate this relationship. Although numerous confounders were accounted for, we cannot entirely exclude the possibility that unmeasured confounders may have influenced our results. For instance, relying on 24-h dietary recall to assess nutrient intake may result in recall bias, potentially compromising data accuracy. Therefore, future studies may consider the use of a combination of assessment methods or the introduction of more objective biomarker tests and other means, which is poised to enhance the dependability and persuasiveness of the outcomes.

## 5 Conclusion

To conclude, our results indicate that total dietary sugar, total fat, and calcium intake is significantly correlated with sleep disorder in hypertensive patients. This study suggests that high sugar, fat, and calcium intake may increase sleep disturbances in hypertensive patients.

## Data Availability

The original contributions presented in the study are included in the article/supplementary material, further inquiries can be directed to the corresponding author.
